# Epigenetic gambling & epigenetic drift as potential mechanisms underlying the quasi-stochastic distributions of late life neurodegenerative disorders

**DOI:** 10.1186/1750-1326-7-S1-L20

**Published:** 2012-02-07

**Authors:** George M Martin

**Affiliations:** 1 Departments of Pathology & Genome Sciences, University of Washington, Seattle, Washington, USA

## Background

Generations of biogerontologists have repeatedly observed striking variations in lifespans among individual members of genetically identical cohorts from a wide variety of species when aged under apparently identical environments. It is a reasonable assumption that these differences are accompanied by contrasting patterns of healthspan. Recent studies with *C. elegans* provide strong evidence that these lifespan variations are largely non-heritable (SL Rea et al., Nature Genet37:894, 2005). One can therefore, conclude that they are driven by stochastic events.

## Methods

The frequencies of somatic mutation seem insufficient to explain these variations. They seem likely to be epigenetically based. I have suggested an explanation (GM Martin, Aging Cell 8:761, 2009) that can be viewed as an extension of the antagonistic pleiotropic model of aging proposed by the late George C. Williams (Evolution 11: 398, 1957). In brief, it is proposed that variegated gene expression within families of homologous cells was selected by evolution as an adaptive trait to ensure survival within unpredictable ecologies.

## Results

Species that evolved within ecologies with high degrees of environmental challenges were thought to have developed more marked degrees of “epigenetic gambling” than those that evolved under more predictable ecologies. Once established, however, variable degrees of “epigenetic drift” accompany aging, resulting in non-adaptive pathophysiology that escapes the force of natural selection. One would therefore predict that species with higher degrees of initial epigenetic gambling would exhibit shorter lifespans. Evidence for such epigenetic drift has been established among human identical twins (MF Fraga et al., PNAS 102:10604, 2005) and inbred mice (R Bahar et al., Nature 441:1011, 2006). This scenario could provide an explanation for the quasi-stochastic distributions of the lesions of a variety of neurodenerative disorders, including forms of Parkinson’s disease, frontotemporal dementias and dementias of the Alzheimer type (DAT). For the case of DAT, for example, one can envisage “a perfect storm” whereby epigenetic drifts have resulted in cell foci having exceptionally low levels of alpha secretase, neprilysin and insuling-degrading enzyme while, at the same time, developing exceptionally high levels of beta secretase, gamma secretase and gamma secretase activating enzyme.

## Conclusion

The theory also predicts analogous pathogenetic mechanisms for a wide variety of geriatric disorders, including benign and malignant neoplasms, in which the first step in oncogenesis could be an escape from proliferative homeostasis leading to hyperplastic clones. Support for this idea comes from studies of expansions of clones with neutral mutations surrounding adenocarcinomas of the colon (JJ Salk et al., PNAS 106:20871, 2009).

